# Cryptosporidium-Induced Myocarditis: A Unique Case Study

**DOI:** 10.7759/cureus.95559

**Published:** 2025-10-28

**Authors:** Fatima Eltayeb, Emily Rawlinson, Raymol Keelan

**Affiliations:** 1 Acute Medicine, Wythenshawe Hospital, Manchester, GBR

**Keywords:** cardiac magnetic resonance imaging, case report, cryptosporidium, gastroenteritis, myocarditis

## Abstract

Myocarditis is the inflammation of the myocardium. While viral infections commonly cause it, we present a unique case of *Cryptosporidium*-induced myocarditis in an immunocompetent individual. A 33-year-old man presenting with acute chest pain and loose stools was found to have troponin levels of over 1,000 ng/L. Initial viral screen tests were negative, and an inpatient echocardiogram showed an insignificant rim of pericardial effusion. Computed tomography of the coronary arteries showed no evidence of coronary artery disease. He was later discharged with outpatient cardiac magnetic resonance imaging (MRI), which subsequently showed acute focal sub-epicardial fibronecrosis, consistent with acute myocarditis. Following this, stool cultures were found to be positive for *Cryptosporidium hominis*, a protozoan parasite. After a further review of the history, it was discovered that the patient had recently been exposed to dead pigeons and faeces at a workplace construction site. This rare presentation demonstrates the importance of considering less common infectious agents even in immunocompetent patients, as there is only one other case study ever published. Furthermore, it highlights the increasing value of using cardiac MRI as a non-invasive investigation for myocarditis.

## Introduction

Acute myocarditis is inflammation of the heart of recent onset (usually less than one month) [1]. The aetiology of myocarditis can be divided into infectious and non-infectious, including autoimmune and drug causes. While viral infections are the most common infectious cause [2], rarely, parasites have been identified as the source.

*Cryptosporidium *is a protozoan that infects the gastrointestinal tract, causing acute gastroenteritis. The transmission is primarily through the faecal-oral route via contaminated water sources or infected animals [3]. The exact mechanism by which this parasite can reach the heart is currently unclear, but a possible haematogenous spread has been suggested [4]. Here, we present an unusual case of *Cryptosporidium-*inducing myocarditis in an immunocompetent patient.

## Case presentation

A 33-year-old male with no past medical history presented to the emergency department with a five-day history of diarrhoea, fever, and chest pain. An electrocardiogram (ECG) showed non-dynamic mild inferolateral ST elevation, and initial blood tests showed no abnormalities other than a C-reactive protein level of 32 mg/L. It was believed that this was likely gastroesophageal reflux disease and was initially discharged from the emergency department with safety-netting advice. A few hours later, he was called back to the emergency department when blood tests showed an elevated troponin level at 1,054 ng/L (reference range: 0-14 ng/L).

A further history illustrated that he had been passing watery, yellow stools and had recently eaten a takeaway. He was a builder who had a history of recreational cocaine use and had last used a week prior to admission. The timing of this was not believed to be in keeping with cocaine-induced myocarditis. Initial viral screen was negative for human immunodeficiency viruses (HIV), hepatitis B, and hepatitis C.

He was admitted to the acute medical ward, and a repeat serum troponin was 1,360 ng/L. In light of the high troponin level and presumed viral symptoms, it was treated as myopericarditis, and stool cultures were sent to exclude *Campylobacter*. The same day, further viral screens were sent, which excluded more common causes of myocarditis and gastroenteritis, including adenovirus, rotavirus, and norovirus. Other viral agents were excluded through nose and throat viral swabs that underwent polymerase chain reaction (PCR) testing. These included coronavirus, influenza A and B, and respiratory syncytial virus. An inpatient echocardiogram was performed, which showed normal function and an 'insignificant rim of pericardial effusion 0.8 cm, with no evidence of tamponade'. Following this, he underwent a computed tomography of the coronary arteries, which showed no evidence of coronary artery disease. He was discharged home for an outpatient cardiac magnetic resonance imaging (MRI) scan and symptomatic treatment, including bisoprolol 1.25 mg daily, as this has been shown to improve outcomes, although this is debated in the absence of left ventricular dysfunction [5]. The European Society of Cardiology advises beta blockers to improve symptoms and prevent arrhythmias [6].

Two weeks later, he returned to the same-day emergency care unit to review the stool samples taken at the time of admission. PCR detected *Cryptosporidium hominis*. The patient had no recent travel history and no contact with farm animals, but had recent workplace exposure to many dead pigeons and faeces while power washing the roof of an apartment building. Faeco-oral route, which could be transmitted through unwashed hands, for example, was presumed to be the likely source of the infection. Repeat blood tests were reassuring with a troponin level of 4 ng/L with normalised inflammatory markers, in addition to his symptoms being much improved.

**Figure 1:**
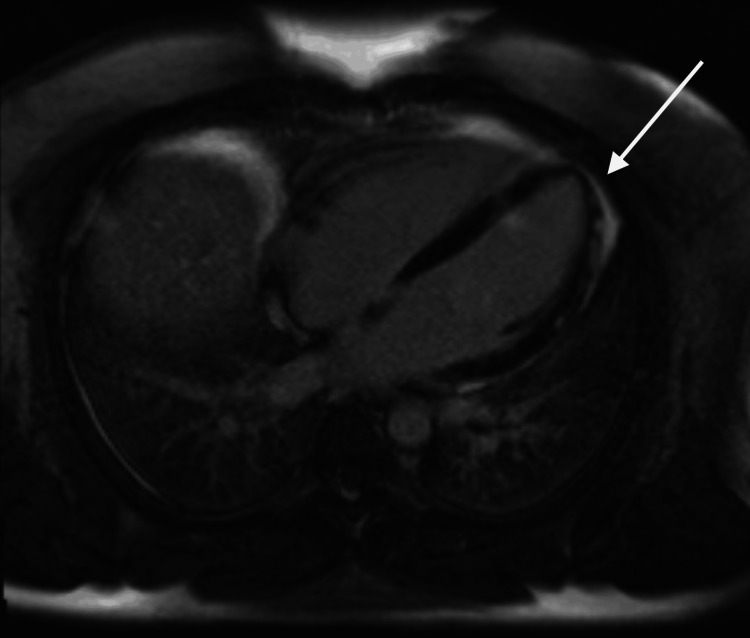
Cardiac magnetic resonance imaging showing fibronecrosis and oedema

Due to the association of *Cryptosporidium *infection and immune incompetence, repeat HIV testing was performed, which was also negative. The cardiac MRI performed 12 days after the first presentation showed acute focal sub-epicardial fibronecrosis in mid-apical lateral and apical inferior left ventricle, as well as adjacent oedema (Figure 1), with normal ventricular function, consistent with recent acute myocarditis. The normal contractile function was a favourable prognostic sign, and a full recovery was expected. The patient was discharged after receiving advice to avoid strenuous activity or heavy alcohol consumption, with follow-up with cardiology and a plan for repeat cardiac MRI. In light of the occupational exposure to this disease, Public Health England was notified for tracking purposes.

## Discussion

Acute myocarditis is a potentially life-threatening condition and can often present itself as a range of symptoms, including chest pain, heart failure, and palpitations [7]. The pathophysiology behind it is not well understood, but there have been many theories, including direct invasion of myocardial tissue, immune-mediated responses, and molecular mimicry [8]. Myocarditis is most often associated with viral infections; however, rarely, parasites have been identified [9]. Further research into the pathophysiology of infectious causes of myocarditis, particularly parasites, can aid in establishing causality, as current literature is limited.

*Cryptosporidium *is a well-known cause of gastroenteritis, and in the immunocompetent host, it usually presents as a short-term, diarrheal illness that self-resolves within a few weeks to a month [10]. It more commonly presents, however, as an opportunistic infection in the immunocompromised patient [10]. As such, it is important to consider investigating HIV/AIDS (acquired immune deficiency syndrome) in patients testing positive for *Cryptosporidium*. Commonly, this infection is linked to contaminated water sources or animal contact, with annual cases confirmed through laboratory testing in the UK totalling between 3.8 and 11.9 per 100,000 population between 2014 and 2023 [11].

There have been reports of extraintestinal manifestations of this condition in the respiratory tract and biliary tree [4,10,12]. Our literature review has identified only one prior documented case of *Cryptosporidium-*associated myocarditis [13]; therefore, this case adds to the growing literature on extraintestinal manifestations [4] and highlights gaps in research. Further research into parasite-induced myocarditis, including *Cryptosporidium*, can aim to identify pathophysiology and causality, to propose new treatment strategies, and better predict prognosis. Clinicians should therefore be aware of the possibility of these extra-intestinal manifestations and widen the differential diagnosis in such circumstances. A thorough history to identify possible exposures is vital.

While traditionally endomyocardial biopsy has been the standard diagnostic test for myocarditis, due to the potential risks, there has been an increased reliance on cardiac MRI as a non-invasive investigation [6,7]. In this case, the risks outweighed any potential benefits, and therefore the biopsy was forgoed. This represents a limitation in establishing causality; however, given that several other infectious causes were excluded and the systematic review revealed no features suggestive of an autoimmune condition or likely drug causes, it was deemed the most probable causative agent.

The management of infectious causes of myocarditis is usually supportive while treating any associated complications, such as arrhythmias or heart failure. Prognosis is generally favourable if ECG changes resolve, left ventricular function is normal, and no regional wall motion abnormalities are detected [6]. Strenuous exercise should be avoided following an episode to prevent the small risk of a potentially fatal arrhythmia. There has been little to no benefit evidenced with the use of immunosuppressive agents [2].

The management of cryptosporidiosis is also supportive in the immunocompetent host. If there are persistent symptoms, nitazoxanide, an anti-protozoal, can be considered according to national guidelines [3]. In the immunocompromised, the first line of management is treating the primary disorder, as usually improvement is elicited with improvement of the underlying condition. The use of nitazoxanide is not approved to treat immunocompromised patients as it has not been proven to be superior to the placebo [3].

## Conclusions

This case shows a rare cause of acute myocarditis, highlighting the importance for clinicians to remain open-minded when dealing with atypical presentations and to consider rarer infectious causes, particularly when there is positive exposure history. As there has only ever been one other case report of *Cryptosporidium*-induced myocarditis, it remains unclear whether it is caused by direct invasion of the myocardium or perhaps a secondary immune response. While the overall prognosis is good for acute myocarditis, due to the scarcity of its association with *Cryptosporidium*, it is difficult to draw firm conclusions about the prognosis of *Cryptosporidium*-induced myocarditis. In this case, preserved cardiac function was a good prognostic sign.
